# Variability of bile bacterial profiles and drug resistance in patients with choledocholithiasis combined with biliary tract infection: a retrospective study

**DOI:** 10.1093/gastro/goae010

**Published:** 2024-02-22

**Authors:** Hang Zhang, Yuchen Cong, Lichao Cao, Kuijin Xue, Peng Qi, Qingdong Mao, Cong Xie, Yushan Meng, Bin Cao

**Affiliations:** Department of Gastroenterology, The Affiliated Hospital of Qingdao University, Qingdao, Shandong, P. R. China; Department of Medicine, Qingdao University, Qingdao, Shandong, P. R. China; Department of Gastroenterology, The Affiliated Hospital of Qingdao University, Qingdao, Shandong, P. R. China; Department of Medicine, Qingdao University, Qingdao, Shandong, P. R. China; Department of Radiology, Baylor University, Waco, TX, USA; Department of Gastroenterology, The Affiliated Hospital of Qingdao University, Qingdao, Shandong, P. R. China; Department of Gastroenterology, The Affiliated Hospital of Qingdao University, Qingdao, Shandong, P. R. China; Department of Gastroenterology, The Affiliated Hospital of Qingdao University, Qingdao, Shandong, P. R. China; Department of Gastroenterology, The Affiliated Hospital of Qingdao University, Qingdao, Shandong, P. R. China; Department of Medicine, Qingdao University, Qingdao, Shandong, P. R. China; Department of Gastroenterology, The Affiliated Hospital of Qingdao University, Qingdao, Shandong, P. R. China; Department of Medicine, Qingdao University, Qingdao, Shandong, P. R. China; Department of Gastroenterology, The Affiliated Hospital of Qingdao University, Qingdao, Shandong, P. R. China

**Keywords:** pathogen, endoscopic retrograde cholangiopancreatography, choledocholithiasis, biliary tract infection

## Abstract

**Background:**

Biliary tract infection is a common complication of choledocholithiasis. This study aimed to analyse the distribution of pathogenic bacteria in bile cultures from patients with choledocholithiasis combined with biliary tract infection to guide clinical application of antimicrobials and reduce the emergence of drug resistance.

**Methods:**

A total of 880 patients were enrolled in this retrospective study from 30 March 2017 to 31 August 2022 at the Affiliated Hospital of Qingdao University in China. Bile specimens were extracted for microbiological culture under aseptic conditions using endoscopic retrograde cholangiopancreatography. Bacterial culture, strain identification, and antimicrobial susceptibility testing were conducted according to the standard protocol. Baseline data were retrieved from patient files.

**Results:**

Overall, 90.34% (795/880) of bile samples showed positive microbiological results and 37.50% (330/880) demonstrated polymicrobial infections. Among the 795 bile specimens with positive culture results, 1,216 pathogenic bacteria were detected, with gram-negative bacilli accounting for 56.33%, gram-positive cocci for 41.86%, and fungi for 1.81%. The predominant gram-negative bacilli in the bile cultures were *Escherichia coli* (30.43%) and *Klebsiella pneumoniae* (13.98%), whereas the main gram-positive cocci were *Enterococcus faecium* (14.04%) and *E. casseliflavus* (4.28%). The annual trend analysis revealed a gradual decrease in the proportion of gram-negative bacilli and a gradual increase in the proportion of gram-positive cocci, with a concomitant decrease in the dominance of *E. coli*. Both *E. faecium* and *E. coli* showed high resistance to conventional antibiotics but high sensitivity to piperacillin/tazobactam, carbapenems, amikacin, and vancomycin.

**Conclusions:**

A significant change has occurred in the bile bacterial spectrum in patients with choledocholithiasis and biliary tract infection. The incidence of gram-positive cocci infections has increased annually, while that of gram-negative bacilli and *E. coli* infections has decreased. Antibiotic administration should be tailored based on the local bacterial profile.

## Introduction

Biliary tract infection (BTI) is a common and frequently occurring disease in clinical practice and is also one of the common causes of acute abdomen pain [1]. The most frequent causes of acute BTI include stones, tumors, parasites, benign bile duct strictures, and biliary surgery, of which choledocholithiasis is the most common factor [2]. Severe BTI can lead to systemic inflammatory response syndrome and multiple organ dysfunction [3–5]. Critical to the successful treatment of BTI is effective antimicrobial therapy and unobstructed biliary drainage [6].

BTI treatment relies heavily on an accurate and comprehensive understanding of the pathogens responsible for the infection. Knowledge of the bacterial spectrum in bile not only facilitates better selection of antibiotic regimens, but also guides the development of individualized treatment strategies and improves treatment outcomes [7, 8]. Previous studies have found that gram-negative bacteria are the most common BTI pathogens, with *Escherichia coli* and *Klebsiella pneumoniae* predominating [9–12]. However, the previous studies were conducted a long time ago and had a small number of samples, making them no longer applicable to the current situation of bacterial infections in the biliary tract. Recent studies have shown that some patients with BTI cannot be effectively treated with conventional antibiotics, as available data and guidelines recommended [13, 14]. As of now, it remains uncertain whether the bacterial spectrum of BTI has evolved or whether there has been an increase in drug resistance rates. This situation poses challenges for the diagnosis and treatment of BTI. Therefore, this necessitates a thorough exploration of changes in the bacterial spectrum and drug resistance profile of BTI for clinical application.

This study aimed to analyse the distribution of pathogenic bacteria in bile in patients diagnosed with choledocholithiasis combined with BTI and thus provide guidance for clinical application of antimicrobials and reduce the emergence of drug resistance. To investigate changes in biliary tract bacterial profile and drug resistance, we pooled the bacterial profiles of bile cultures from these patients over a 6-year period. Additionally, we performed an annual trend analysis to specifically compare the dynamics of the bacterial profiles of the bile cultures.

## Materials and methods

### Study population

This retrospective study was conducted at the Affiliated Hospital of Qingdao University (Shandong, China) between 30 March 2017 and 31 August 2022. The inclusion criteria for the patients were as follows: (i) diagnosed with choledocholithiasis confirmed by using computed tomography (CT), enhanced CT, or magnetic resonance cholangiopancreatography of the epigastrium; (ii) met the diagnostic criteria for BTI; (iii) took endoscopic retrograde cholangiopancreatography (ERCP) treatment; (iv) underwent preoperative examinations, such as cardiac ultrasound, CT, amylase, liver and kidney function, and coagulation function; (v) provided informed consent (from themselves or their families) before ERCP.

**Figure 1.**
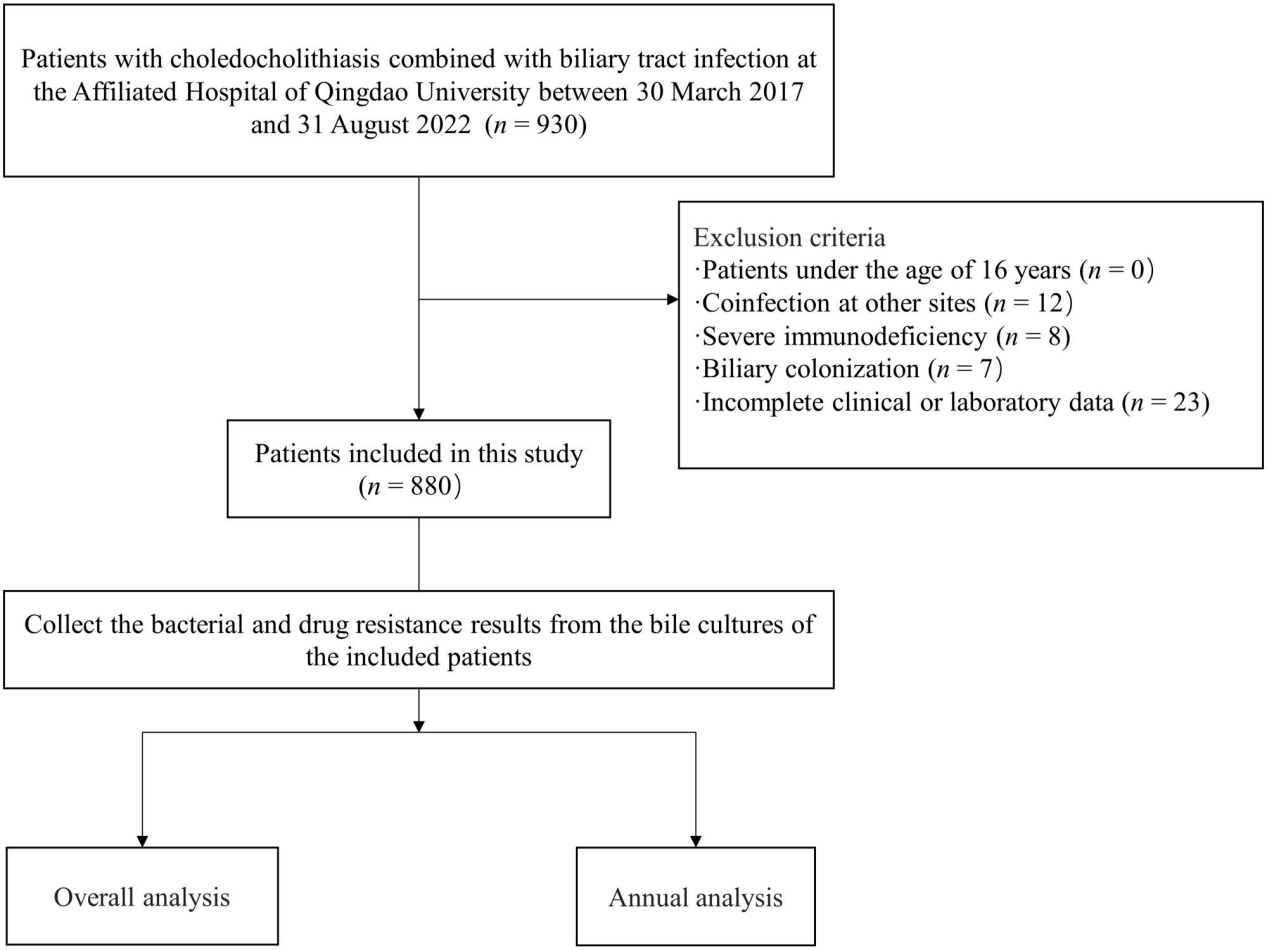
Study flowchart.

The exclusion criteria for the patients were as follows: (i) aged <16 years; (ii) co-infection at other sites; (iii) severe immunodeficiency; (iv) biliary colonization; (v) incomplete clinical or laboratory data (including missing medical records, failure to obtain intraoperative bile for culture, and contaminated specimens) (Figure 1).

Treatment records were reviewed. The number of enrollees, positive bile cultures, and polymicrobial infections were counted from 2017 to 2022, and the number of pathogenic bacteria in each year were counted for an annual data comparison. Moreover, the number of pathogenic bacteria in each year were counted to determine the annual data (such as the annual positivity rate = number of positive bile cultures in the current year/number of bile cultures in the current year). The following information was collected: age, sex, underlying disease, symptoms associated with BTI, preoperative antibiotic use, history of ERCP or ballistic minimally invasive surgery, preoperative findings, and imaging findings. In some cases, patients received short-term antibiotic treatment based on their condition before undergoing ERCP. Depending on the patient's clinical presentation and allergy history, the antibiotic regimen typically included intravenous administration of piperacillin-tazobactam, levofloxacin, or cefepime.

For patients who underwent multiple ERCP procedures, we specifically extracted clinical data from the first ERCP performed at our institution. This approach was adopted to ensure methodological consistency throughout our study.

The study was approved by the Ethics Committee of The Affiliated Hospital of Qingdao University (Ethics ID QYFY WZLL27541). The patients and their families provided written informed consent before the ERCP.

### Bile collection and processing

ERCP was performed using the OLYMPUS TJF-260V electronic duodenoscope (Olympus Tokyo, Japan) by a team of four endoscopists each with 10 years of ERCP experience. Adhering to guidelines, the duodenoscope was sterilized before insertion into the descending duodenum. Upon achieving an optimal position with the duodenal papilla, the scope was straightened and both the biopsy orifice and the duodenal papilla were flushed with saline to minimize bacterial contamination from the oral cavity and upper gastrointestinal tract.

After successful cannulation of the bile duct through the papilla, the guide wire was left in the bile duct. Subsequently, the incision knife was retracted through the duodenal papilla and deflated before entering the bile duct. A sterile syringe was used to extract ∼5–10 mL of bile, which was immediately added to prepared aerobic and anaerobic culture bottles. To ensure optimal conditions, bile specimens were sent to the laboratory within 2 h of collection.

### Instruments and reagents

The isolates were identified using VITEK 2 Compact (MERIEUX, France), a fully automated bacterial identification drug sensitivity analyser. The quality control strains were *E. coli* ATCC25922, *Enterococcus faecalis* ATCC29212, and *K. pneumoniae* ATCC700603, all purchased from the Clinical Laboratory Centre of the Ministry of Health. A drug sensitivity test was performed using the Kirby–Bauer paper diffusion (K–B) method. Extended-spectrum beta-lactamases (ESBLs) were quantified using a phenotypic confirmatory combined-disk test. All results were determined according to the Clinical Laboratory and Standards Institute standards. Because other resistance data were not comprehensive, only ESBLs were counted.

### Statistical analysis

Categorical variables are presented as proportions or rates. Data were analysed using SPSS 26.0 and compared using the *χ*^2^ test or Fisher's exact test. All *P*-values were based on a two-tailed test and differences were considered statistically significant at *P *<* *0.05.

## Results

### Clinical characteristics of the patients

A total of 880 patients met the screening criteria: 486 men and 394 women, aged 67.12 ± 13.80 years. The results of bile cultures in these 880 patients indicated a higher likelihood of positive results among patients with purulent bile and those of advanced age. The baseline characteristics of the included patients are shown in Table 1.

**Table 1.** Characteristics of patients with choledocholithiasis combined with biliary tract infection undergoing ERCP with bile culture

Characteristic	Total bile culture (*n* = 880)	Positive bile culture (*n *=* *795)	Negative bile culture (*n *=* *85)	*P*-value
Age, mean ± SD, years	67.12 ± 13.80	67.95 ± 14.25	65.00 ± 13.30	**0.041**
Sex, female, *n* (%)	394 (44.77)	359 (45.16)	35 (45.18)	0.483
Gallbladder removed, *n* (%)	139 (15.80)	121 (15.22)	18 (21.18)	0.152
Combined intrahepatic stones, *n* (%)	27 (3.07)	23 (2.89)	4 (4.71)	0.555
Combined gallbladder stones, *n* (%)	187 (21.25)	162 (20.38)	25 (29.41)	0.053
Purulent bile at ERCP, *n* (%)	346 (39.32)	325 (40.88)	21 (24.71)	**0.004**
Preoperative antibiotics for more than 3 days, *n* (%)	57 (6.48)	49 (6.16)	8 (9.41)	0.118

SD = standard deviation, ERCP = endoscopic retrograde cholangiopancreatography.

### Microbiological characteristics

Among the 795 bile specimens with positive culture results, 1,216 pathogenic strains were isolated, wherein gram-negative bacilli accounted for 56.33% of the isolates, gram-positive cocci accounted for 41.86%, and fungi accounted for 1.81%, and no anaerobic bacteria were detected. The main gram-negative bacilli among the pathogenic bile culture bacteria were *E. coli* (30.43%) and *K. pneumoniae* (12.25%), and the main gram-positive cocci were *E. faecium* (13.98%) and *E. casseliflavus* (4.28%) (Table 2). A total of 317 (26.07%) cases were observed of *Enterococcus* species (spp.).

**Table 2.** Distribution of different organisms in positive bile cultures

Pathogen	*n*	Percentage (%)
**Gram-negative bacilli**	**685**	**56.33**
*Escherichia coli*	370	30.43
*Klebsiella pneumoniae*	149	12.25
*Proteus mirabilis*	32	2.63
*Citrobacter freundii*	17	1.40
*Klebsiella oxytoca*	16	1.32
*Enterobacter aerogene*	13	1.07
*Enterobacter cloacae*	12	–
*Aeromonas caviae*	12	–
*Pseudomonas aeruginosa*	12	–
*Aeromonas hydrophila*	9	–
*Citrobacter koseri*	4	–
*Stenotrophomonas maltophilia*	3	–
*Acinetobacter baumannii*	3	–
*Lactobacillus gasseri*	3	–
*Lactobacillus salivarius*	2	–
*Leuconostoc lactis*	2	–
*Raoultella ornithinolytica*	2	–
Other	24	1.97
**Gram-positive cocci**	**509**	**41.86**
*Enterococcus faecium*	170	13.98
*Enterococcus casseliflavus*	52	4.28
*Streptococcus salivarius*	47	3.87
*Enterococcus faecalis*	42	3.45
*Enterococcus gallinarum*	25	2.06
*Oral Streptococcus*	21	1.73
*Streptococcus vestibularis*	19	1.56
*streptococcus parasanguis*	17	1.40
*Staphylococcus epidermidis*	16	1.32
*Streptococcus mitis*	12	–
*Enterococcus hirae*	12	–
*Streptococcus anginosus*	10	–
*Staphylococcus aureus*	8	–
*Enterococcus avium*	8	–
*Streptococcus cristatus*	8	–
*Actinomyces odontolyticus*	8	–
*Enterococcus raffinosus*	6	–
*Staphylococcus haemolyticus*	5	–
*Streptococcus alactolyticus*	3	–
*Veillonella parvula*	3	–
*Streptococcus constellatus*	2	–
*Streptococcus gordonii*	2	–
Other	13	1.07
**Fungus**	**22**	**1.81**
*Candida albicans*	10	–
*Candida tropicalis*	7	–
*Candida glabrata*	2	–
*Candida krusei*	2	–
*Candida parapsilosis*	1	–

– indicates <1% of the total.

### Annual changes in bacterial profiles

**Figure 2.**
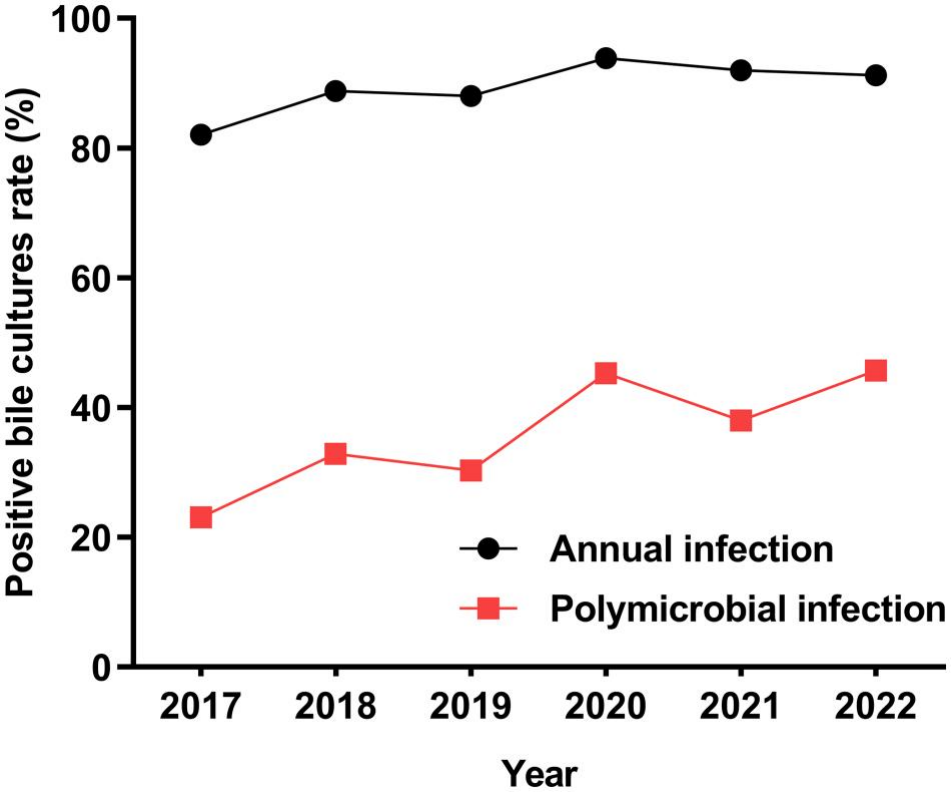
Changes in the rate of positive bile culture and polymicrobial infection.

Among all patients with choledocholithiasis and BTI, the positive bile culture rate was 90.34% (795/880) and the polymicrobial infection rate was 37.50% (330/880) over the 6-year study period. There were 199 cases (22.61%) of two mixed bacterial infections, 110 (12.50%) of three bacterial infections, and 21 (2.39%) of four bacterial infections. The most common type of mixed infection was *E. coli* mixed with *E. faecalis*. The annual positivity rates of bile cultures from 2017 to 2022 were 82.05%, 88.81%, 88.03%, 93.87%, 91.98%, and 91.34%, respectively. The annual positivity rates of polymicrobial infections were 23.08%, 32.84%, 30.28%, 45.28%, 37.97%, and 45.67%, respectively (Figure 2).

**Figure 3.**
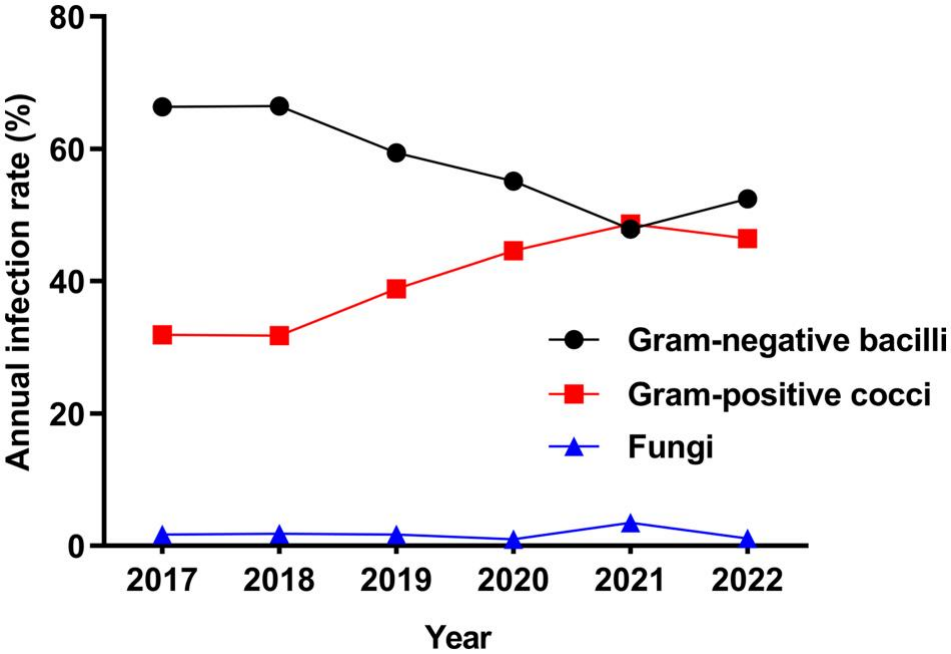
Changes in the positive rate of gram-negative bacilli and gram-positive cocci.

The annual proportion of gram-negative bacilli in the bile cultures from 2017 to 2022 was 66.38%, 66.47%, 59.43%, 55.10%, 47.88%, and 52.46%, respectively; that of gram-positive cocci was 31.91%, 31.74%, 38.86%, 44.59%, 48.65%, and 46.45%, respectively; and that of fungi was 1.72%, 1.80%, 1.71%, 0.96%, 3.47%, and 1.09%, respectively. The annual infection rate of gram-negative bacilli showed a decreasing trend, the infection rate of gram-positive cocci demonstrated an increasing trend, and the fungal infection rate remained low and flat throughout the study period (Figure 3).

**Figure 4.**
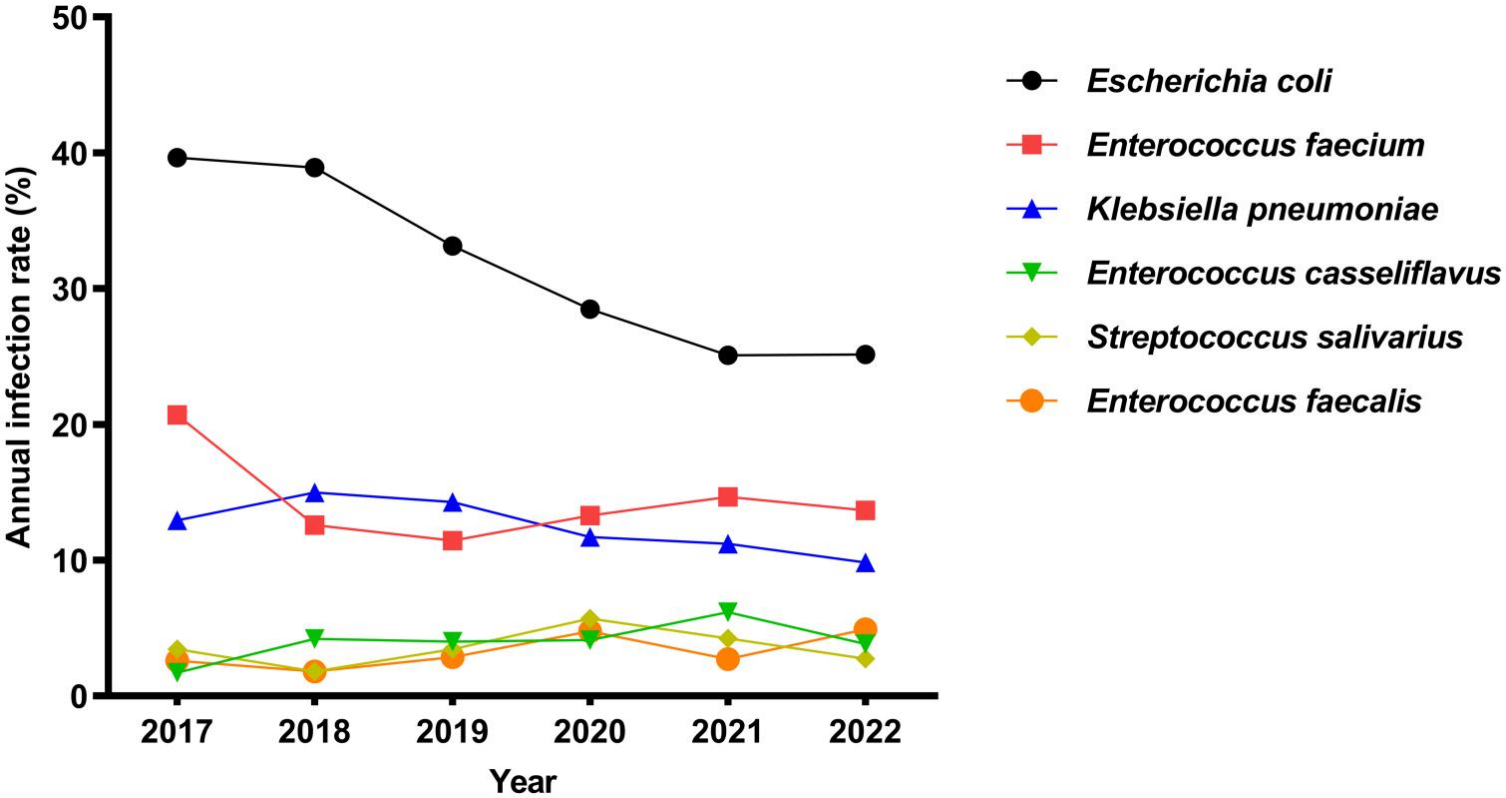
Trend change in the percentage of pathogenic bacteria detected in bile culture.

Data on the primary pathogens across the study period show that the proportion of *E. coli* decreased, whereas that of *E. faecium*, *K. pneumoniae*, *E. casseliflavus*, *Streptococcus salivarius*, and *E. faecalis* remained relatively stable (Figure 4).

### Antibiotic resistance of common pathogenic bacteria cultured from bile

The results of drug resistance tests against antibacterial drugs for common gram-positive and gram-negative bacteria are shown in Tables 3 and 4. *Escherichia coli* and *K. pneumoniae* had the highest sensitivity to tigecycline, followed by carbapenems (imipenem, ertapenem, meropenem) and tobramycin, and higher resistance to cefazolin. *Enterococcus faecium* and *E. casseliflavus* had the highest sensitivity to tigecycline, followed by linezolid and vancomycin.

**Table 3.** Resistance rates of dominant gram-negative bacilli to antimicrobial drugs in bile cultures

Antimicrobial agent	*Escherichia coli* (*n *=* *370)		*Klebsiella pneumoniae* (*n *=* *149)	
	*n*	%	*n*	%
Amoxicillin/clavulanic acid	93	25.14	31	20.81
Amikacin	19	5.14	15	10.07
Ampicillin	204	55.14	77	51.68
Aztreonam	167	45.14	64	42.95
Ceftazidime	115	31.08	55	36.91
Cefazolin	303	81.89	112	75.17
Ciprofloxacin	222	60.00	75	50.34
Ceftriaxone	204	55.14	79	53.02
Cefuroxime	229	61.89	82	55.03
Ampicillin/sulbactam	159	42.97	60	40.27
Cefepime	96	25.95	34	22.82
Gentamicin	181	48.92	75	50.34
Imipenem	4	1.08	4	2.68
Tobramycin	7	1.89	0	0.00
SMZ/TMP	204	55.14	79	53.02
Piperacillin/tazobactam	26	7.03	30	20.13
Ertapenem	4	1.08	0	0.00
Tigecycline	0	0.00	0	0.00
Ciprofloxacin	222	60.00	60	40.27
Cefoperazone/sulbactam	41	11.08	21	14.09
Cefotaxime	248	67.03	86	57.72
Meropenem	7	1.89	6	4.03
**ESBLs**	**163**	**44.05**	**58**	**38.93**

SMZ/TMP = sulfamethoxazole/trimethoprim, ESBLs = extended-spectrum beta-lactamases.

**Table 4.** Resistance rates of dominant gram-positive cocci to antimicrobial drugs in bile cultures

Antimicrobial agent	*Enterococcus faecium* (*n *=* *170)		*Enterococcus casseliflavus* (*n *=* *52)	
	*n*	%	*n*	%
Ampicillin	133	78.24	44	84.62
Ciprofloxacin	127	74.71	40	76.92
Erythromycin	135	79.41	33	63.46
Nitrofurantoin	18	10.59	–	–
Gentamicin-High	61	35.88	1	1.92
Penicillin	153	90.00	44	84.62
Quinupristin and Dalfopristin	35	20.59	13	25.00
Tetracycline	58	34.12	7	13.46
Vancomycin	2	1.18	0	0.00
Linezolid	1	0.59	0	0.00
Moxifloxacin	137	80.59	18	34.62
Tigecycline	0	0.00	0	0.00
Levofloxacin	121	71.18	17	32.69

– indicates data not available; Gentamicin-High: ≥500μg/mL.

## Discussion

This single-centre retrospective study evaluated the microbiological profiles of biliary aspirates collected during ERCP from 880 patients with choledocholithiasis complicated by BTI and performed an annual trend analysis. This led to four conclusions. First, patients with choledocholithiasis and BTI have a high rate of positive bile cultures and polymicrobial infections. Second, bile culture results show that, in the 6-year study period, gram-negative bacilli dominated, and the predominant pathogenic bacteria were *E. coli* and *E. faecium*. Third, the annual trend analysis showed a yearly increase in gram-positive cocci infections, whereas the predominance of gram-negative bacilli (chiefly *E. coli*) gradually decreased. Fourth, common biliary pathogens are highly resistant to conventional antibiotics.

In this study, the positive bile culture rate showed a slowly increasing trend over the 6-year period, exceeding 90% in 2019, 2021, and 2022. These rates are similar to the findings of Gromski *et al.* [15] and Müssle *et al.* [16] and higher than the rates reported by Rupp *et al.* [17]. This difference may be attributed to the different sampling methods and statistical criteria used in different regions. In the present study, we found that older patients are more susceptible to BTIs, possibly owing to older patients having relatively lower gastric acid concentrations, weakened gastrointestinal motility, and more frequent intestinal disorders [18], which increase the probability of intestinal bacteria entering the biliary tract in a retrograde manner. Moreover, older patients often have compromised cellular and humoral immune function due to the decline in immune cell function and the presence of chronic diseases [19]. While guidelines [8] do not recommend the routine use of antibiotics prior to ERCP procedures, antibiotics may be considered for patients with concomitant biliary sepsis, severe immunosuppression, or advanced age. In our study, we found that not using antibiotics or only short-term use prior to ERCP did not exert a notable influence on the positivity rate of bile cultures. This may be attributed to the relatively short duration of antibiotic use and the specific nature of biliary anatomy.

In this study, the number of specimens with polymicrobial infections was 330 and the rate of polymicrobial infections reached 45.67% in 2022. We believe the increasing trend of polymicrobial infections merits further clinical investigation. Currently, studies on polymicrobial BTI and standardized treatment plans for antibiotic treatment of mixed infections are lacking. The China Antimicrobial Resistance Surveillance System analysed 268,016 strains of pathogenic bacteria cultured from bile samples from 2014 to 2019 and reported the predominance of gram-negative bacilli, whose proportion in bile cultures was ∼70% [20]. Previous studies have shown the predominance of gram-negative bacilli in bile cultures, with *E. coli* being the most prominent [12, 13, 16, 21]. In our study, longitudinal analysis of the data from our hospital over the past 6 years revealed a significant change in the bacterial spectrum of BTI, with a decreasing trend in the proportion of gram-negative bacilli and a gradual increase in the proportion of gram-positive cocci. In a recent study of bile cultures by Zhao *et al.* [22], a trend toward an increasing proportion of gram-positive cocci over the past 6 years was found, despite the continued predominance of gram-negative species in the cultures, which is consistent with the results of this study.

In our study, the detection rates of gram-positive and gram-negative bacteria in bile cultures were nearly the same across years from 2020 to 2022, and even higher for cocci than for bacilli in 2021. One possible reason for this change may be that cephalosporin and quinolone antibiotics are being irregularly used in many clinical conditions to inhibit the growth of gram-negative bacilli, resulting in a decrease in the proportion of gram-negative bacilli. Additionally, the use of broad-spectrum antibiotics can induce dysbiosis of the intestinal flora, which may indirectly affect bacterial species in the biliary tract [23–25]. Moreover, invasive biliary medical procedures can increase the risk of migration of intestinal flora into the biliary tract. Karasawa *et al.* [26] showed that patients with a history of endoscopic sphincterotomy (EST), intensive care unit stay, bile duct stenting, and cholecystectomy were prone to intestinal flora migration and that EST was an independent risk factor for biliary enterococcal infection, suggesting that invasive biliary procedures can further contribute to the increased proportion of gram-positive cocci in the bile. In addition, environmental contaminants, such as polycyclic aromatic hydrocarbons, microplastics, and antibiotic residues, may directly or indirectly affect the intestinal flora, leading to changes in the bacterial profile of BTI [27, 28].

The three leading pathogens detected in bile cultures during the study period were *E. coli* (30.43%), *E. faecium* (13.98%), and *K. pneumoniae* (12.25%). Although *E. coli* has consistently been the dominant pathogen in the study results, annual analysis indicates a gradual decrease in its proportion of cases, which is consistent with the annual trend change of gram-negative bacilli. Currently, empirical treatment for BTI primarily targets enterobacteria; however, *Enterococcus* species has emerged as a significant pathogen that cannot be ignored in such infections. In our study, we detected 317 cases (26.07%) of *Enterococcus* spp. infection. This finding highlights the importance of considering *Enterococcus* in the selection of empirical treatment for BTIs. A study on patients with BTI showed that *Enterococcus* accounted for ≤67.7% of biliary pathogens [15]. Empirical antibiotics may not target *Enterococcus*, which can lead to poor treatment outcomes.

On analysing the data on bacterial drug resistance from 2017 to 2022, the dominant pathogenic bacteria are generally found to be resistant to common antibiotics. For instance, the detection rates of ESBLs in *E. coli* and *K. pneumoniae* are reported as 44.05% and 38.93%, respectively. This underscores the need to closely consider the drug resistance profiles of BTI pathogens. Because ESBLs catabolize penicillin and cephalosporin [29, 30], the detection of ESBLs in bile pathogens is critical for empirical drug selection. Research conducted by Suh *et al.* [31] revealed a significant increase in antibiotic resistance among common pathogens in the biliary tract over time. The study by Zhao *et al.* [22] on the microbiology of BTI found that the rates of ESBLs positivity were 52% for *E. coli* and 21% for *K. pneumoniae*. Furthermore, Jang *et al.* [2] discovered that the prevalence of BTIs caused by ESBL-producing organisms has increased during the last 10 years. These variations in resistance could be attributed to varying medication practices, healthcare conditions, medical standards, and economic development levels across different regions. Penicillin and cephalosporin should be used cautiously in the treatment of patients infected with pathogenic ESBLs. Carbapenems and β-lactamase inhibitor combinations may be used if necessary. When facing ESBL-producing organisms, the guidelines [8] recommend considering the use of carbapenems, piperacillin/tazobactam, and aminoglycosides. These medications not only possess excellent biliary penetration characteristics, but also effectively combat the infecting strains, thereby providing better treatment for patients with BTI [32].

This study included 880 patients with choledocholithiasis combined with BTI. The large sample size increased the reliability of the results and revealed significant trends in the spectrum of pathogenic organisms in recent years, providing a valuable clinical reference. This study has several limitations. This was a retrospective study; therefore, we could not obtain sufficient information regarding the preoperative antibiotics administered to patients. It is possible that a small proportion of patients who underwent ERCP did not undergo bile aspiration, which may have introduced a selection bias. The single-centre retrospective nature of this study made it prone to be influenced by regional differences, being one-sided, and having other limitations. Biliary pathogens, especially multidrug-resistant bacteria, have a corresponding clinical and national economic impact. In the future, we intend to conduct a larger prospective multicentre study to investigate changes in the bacterial profiles of bile cultures to guide the optimal clinical use of antimicrobial drugs and reduce the emergence of drug-resistant bacteria.

In conclusion, the bile culture results from the past 6 years were dominated by *E. coli*, but the bacterial spectrum was found to have changed significantly. The proportion of *E. coli* showed a decreasing trend by the year, whereas that of gram-positive cocci increased significantly, which is substantial for guiding the clinical management of BTI. For patients with choledocholithiasis and BTI, timely and reasonable use of relevant antibiotics (in accordance with drug sensitivity considerations) is key to effective treatment following adequate biliary drainage. Therefore, antibiotics should be administered according to the specific infection profiles of patients, with minimal use of broad-spectrum antibiotics.

## Authors’ Contributions

H.Z. and Y.C. conceived of and designed the project; L.C., K.X., P.Q., Q.M., C.X., and Y.M. collected and analysed the data; H.Z. and B.C. drafted the manuscript. All authors read and approved the final manuscript.
